# Secular Trends of Mortality and Years of Life Lost Due to Chronic Obstructive Pulmonary Disease in Wuhan, China from 2010 to 2019: Age-Period-Cohort Analysis

**DOI:** 10.3390/ijerph191710685

**Published:** 2022-08-27

**Authors:** Yan Guo, Jianjun Bai, Xiaoxia Zhang, Qiman Jin, Yijun Liu, Chuanhua Yu

**Affiliations:** 1 Wuhan Center for Disease Control and Prevention, Wuhan 430022, China; 2 School of Public Health, Wuhan University, Wuhan 430071, China

**Keywords:** age-period-cohort model, mortality, years of life lost, Wuhan, temporal trends

## Abstract

Background: Chronic obstructive pulmonary disease (COPD) has been an important public health issue in China. This study aimed to analyze the temporal trends in mortality and years of life lost (YLL) from COPD, and explore the effects of age, period, and cohort in Wuhan, China from 2010 to 2019. Methods: Data were collected from the cause of death surveillance system in Wuhan. Age-standardized mortality rate (ASMR), age-standardized YLL rate (ASYR) and the estimated annual percent changes (EAPC) were calculated to evaluate the temporal trends. The age-period-cohort (APC) model was adopted to estimate the age, period, and cohort effects. Results: From 2010 to 2019, COPD accounted for 26,051.15 deaths and 394,659.58 person years YLL in Wuhan. Recently, the death burden of COPD in Wuhan has somewhat improved, especially after 2015, with declining trends in ASMR and ASYR. Additionally, the ASMR and ASYR of COPD was higher in males. And these of males showed the overall upward trends, with EAPCs of 1.06 (0.13, 2.00) and 1.21 (0.12, 2.31), respectively, while females showed downward trends since 2010. According to APC model, the age effect of COPD increased with age, and the cohort risk ratios (RRs) followed the overall downward trends. Period RRs for the Wuhan population generally tended to rise and then fall, with females showing a clear downward trend after 2015, while period RRs for males maintained an upward trend throughout the study period. Conclusions: Recently, the death burden from COPD in Wuhan has improved, especially after 2015, with improvements in ASMR, ASYR and period RRs. Sex differences still exist. COPD posed a greater threat to the elderly, especially males. Public health managers should continue to execute more targeted programs to lessen the death burden of COPD in Wuhan.

## 1. Introduction

With urbanization, aging, environmental and lifestyle changes, non-communicable diseases (NCDs) have become the main contributors to disease burden worldwide [1,2]. As a leading type of NCDs, chronic obstructive pulmonary disease (COPD), characterized by high morbidity, mortality, long duration and high cost of treatment, has led to 3.28 million deaths and 54.59 million years of life lost (YLL) worldwide in 2019 [3]. Although the global COPD mortality and YLL rates have generally trended downward from 2010, notably, approximately 90% of COPD deaths have occurred in low- and middle-income countries, which posed a significant public health challenge to global policymakers [4,5].

China—the largest developing country—has undergone rapid economic growth and experienced an increased average life expectancy and substantial changes in lifestyle. In 2017, COPD was the third leading cause of death in China, with 970,000 deaths attributed to COPD, posing a substantial healthcare burden in China [6,7]. Meanwhile, a huge population size, rapid aging, relatively large smoking population, and regional and sex disparities have made the improvement of respiratory diseases particularly challenging in China.

Wuhan is one of the largest cities in China, located in central China, with an area of 8594 square kilometers and a population of over 12.32 million. In recent decades, governments have made great efforts to improve people’s respiratory health, including health-care reform, new rural cooperative medical system, COPD healthcare cost reform, air pollution and tobacco control. Hence, it is necessary and urgent to update the status of COPD mortality in Wuhan and better understand the gap between current conditions and expectations. In addition, the YLL is also an important and common burden indicator, taking the death count and life expectancy at death into consideration, which would help to set priorities for prevention and compare the premature mortality experience between different populations [8].

Studying spatial–temporal trends in COPD (mortality and YLL rate) can be helpful in identifying the background factors contributing to variations and can provide an indication based and regional intervention of public health policy. Previous analyses have focused on mortality and YLL rate trends over time, but ignored the accumulation of health risks since birth. In this regard, the age–period–cohort (APC) model is a common sociological, demographic, and epidemiological model utilized to evaluate the age, period, and cohort effects independently on disease mortality and YLL rate [9]. It could decompose the information on mortality or YLL rate into the current death risk faced by the population (age and period effects) and the health risk accumulated since birth (cohort effects). In this study, we adopted the death data from the Wuhan Center for Disease Control and Prevention (CDC) to examine the secular trend of mortality and YLL rate in COPD and assessed the effect of age, period, and birth cohort using the APC model, stratified by sex and age group. The findings of this study may provide new evidence of influencing factors for COPD and help public health managers assess the impact of previous interventions and formulate further policies.

## 2. Materials and Methods

### 2.1. Data Sources

Data for this study were obtained from the cause of death surveillance system for the resident population in Wuhan from 2010 to 2019, which is a population-wide surveillance system covering almost 100% of the population in Wuhan. The death certificates of residents are issued by relevant medical institutions and directly reported through the Internet system in a timely manner for quality review by the CDC. The causes of death are coded according to the 10th edition of the International Classification of Diseases (ICD-10) (J41–J42.4, J43–J44.9). Generally, a death report case database is considered as qualified if its resident population mortality or standardized resident population mortality reach 3‰. The population data of different sexes and age groups in Wuhan and 13 districts over the past decade were provided by the Wuhan public security department.

Deaths, YLLs, mortality, and YLL rates are all the important indicators in death burden studies. COPD death data from 13 districts were adjusted according to the under-reporting rates with logical verification and garbage code assignment, based on the SAS program developed by the Chinese CDC. Garbage codes are those that cannot or should not be considered as the underlying cause of death, such as mis-assigned codes, inaccurate coding, misclassification, etc. Mortality and YLL rates were calculated based on the adjusted data. We adopted the abbreviated life table to generate the life expectancy and cause-eliminated life expectancy (CELE). CELE means that people who would have died from a specific disease would live longer if this death cause had been eliminated [10]. Thus, the increase in life expectancy (CELE - life expectancy) could reflect the severity of the health impact of a given death cause on the population. Years of life lost (YLLs) is a metric of premature death calculated as the sum of each death multiplied by the standard life expectancy at each age [8].

### 2.2. Statistical Analysis

We adopted the age-standardized rate (ASR) and estimated annual percentage change (EAPC) to quantify the trends of COPD mortality and YLL rate. Standardization is necessary when comparing several populations with different age structures or for the same population over time in which the age profiles change accordingly. The age-standardized mortality and YLL rates (per 100,000) were calculated through direct age standardization with the Chinese sixth census in 2010 as the standard population.

$$ASR=\frac{\sum_{i=1}^{A}a_{i}w_{i}}{\sum_{i=1}^{A}w_{i}}\times 100,000$$

where *a_i_* is the age-specific rates of *i*th age group; *w_i_* is the number of people in the same age group *i* of the Chinese sixth census standard population.

Generally, the ASR trend can serve as a good proxy for evaluating the change in disease pattern and the effectiveness of the current prevention strategies within a population. The EAPC is widely used to quantify the trends of ASR of COPD during a period. Both the EAPC and the lower bound of its 95% CI were both >0, indicating an increasing trend of ASR; both the estimated EAPC and the upper bound of its 95% CI were <0, indicating a decreasing trend; otherwise, ASR is considered stable.

Mortality and YLL rates do not just reflect the population’s health risks in a given year, but also the accumulation of health hazards since birth. Traditional analysis would not appropriately quantify these cumulative death/health risks. We adopt the APC model to further evaluate the effect of age, period, and birth cohort on the mortality and YLL rate of COPD [11,12]. The age effect means that different age groups have a different risk of disease. The period effects represent variations in different screening methods, medical technology applications, or disease classification standards over specific periods, which can affect all age groups in a population simultaneously. Cohort effects refer to the impact of some disease determinants that emerge in the early stages of life and accumulate gradually over time, which may be related to lifestyle, external environment, and risk factors exposure.

In the APC model, the age and period intervals must all be equal, and we excluded the ≥85 age group, because the population over 84 years old was assigned as one age group. Thus, the data were categorized into consecutive 5-year periods from 2010 to 2019, successive 5-year age groups from 20–24 years of age to 80–84 years of age, and the corresponding consecutive 14 birth cohorts (1930–1934, 1935–1939, …, 1995–1999) [13,14]. Since the three parameters of the APC model have exact linear dependency (period = age + cohort), we adopted the “two-factor model” from the Epi package (v.2.44) in the R software to address the multicollinearity problem. Wald chi-square tests were conducted to verify the significance of estimable parameters and functions (Supplementary Table S1).

SAS (v.9.4; SAS Institute, Cary, NC, USA) and Python (v.3.7; Python Software Foundation, Wilmington, DE, USA) were used for data processing. R (v.4.0.2; R Foundation for Statistical Computing, Vienna, Austria) for data analysis and APC model. Statistical significance was considered when a two-sided *p* < 0.05.

## 3. Results

### 3.1. Spatial–Temporal Patterns of COPD Burden

**Figure 1:**
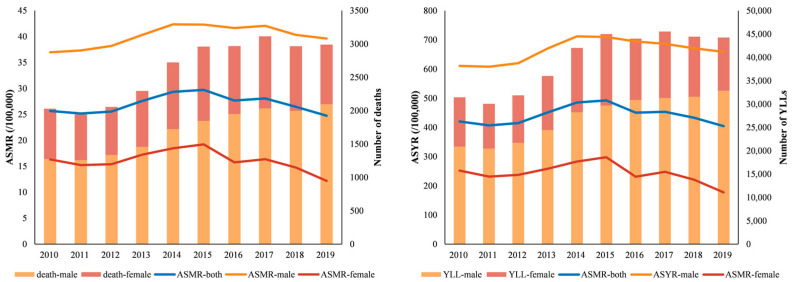
The trends of ASMR and ASYR in Wuhan from 2010 to 2019.

From 2010 to 2019, COPD accounted for 26,051.15 deaths, and 394,659.58 person years YLL in Wuhan. The number of COPD deaths increased by 47.28%, from 2030.65 in 2010 to 2990.78 in 2019. Additionally, YLLs for COPD increased from 31,446.70 to 44,248.42 person years from 2010 to 2019 (Figure 1). Table 1 indicated the details of ASMR, ASYR and EAPC for Wuhan and its 13 districts. The ASMR and ASYR in Wuhan showed the increasing trends until 2015 and decreasing trends thereafter. During the study period, there were differences in the trends of ASR between males and females. The ASMR and ASYR for COPD were higher in males than in females. In addition, compared to males, both ASMR and ASYR showed a relatively significant downward trend in females from 2010 to 2019, while the males showed an overall upward trend with EAPCs of 1.06 (0.13, 2.00) and 1.21 (0.12, 2.31), respectively.

**Figure 2:**
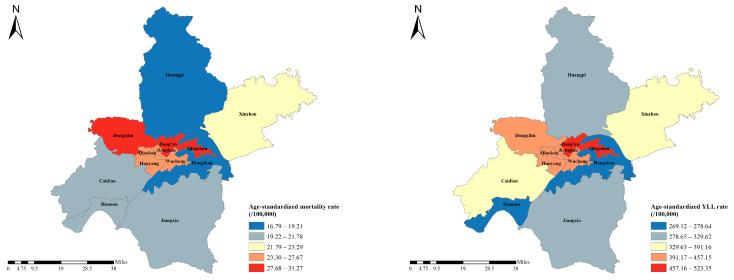
The geographical distribution of ASMR and ASYR in Wuhan in 2019.

Figure 2 showed the geographical distribution of ASMR and ASYR in Wuhan in 2019. Among the 13 districts in Wuhan, the top three with the highest ASMR were Qingshan, Dongxihu and Jiangan, with ASMRs of 31.27 (27.53, 36.96), 30.66 (24.96, 38.58) and 29.76 (26.72, 33.93), respectively. From 2010 to 2019, the ASMR of Hannan, Hongshan and Jiangxia decreased significantly (Table 1). While Wuchang is the only district showing an upward trend of ASMR, with an EAPC of 4.62 (1.93, 7.37). As for ASYR, Qingshan, Jiang’an and Jianghan were the three districts with the highest ASYR, with values of 523.35 (506.75, 541.47), 501.97 (488.53, 516.34) and 457.15 (440.98, 474.71), respectively. Similarly, Hannan, Hongshan and Jiangxia also showed the most obvious declines in ASYR, with EAPCs of −10.97 (−14.62, −7.15), −6.34 (−10.8, −1.65) and −4.96 (−8.71, −1.06), respectively. Additionally, Wuchang also showed the increasing trend in ASYR, with an EAPC of 4.90 (1.58, 8.32) (Table 1).

### 3.2. Life Expectancy and COPD Burden by Ages and Sex

**Figure 3:**
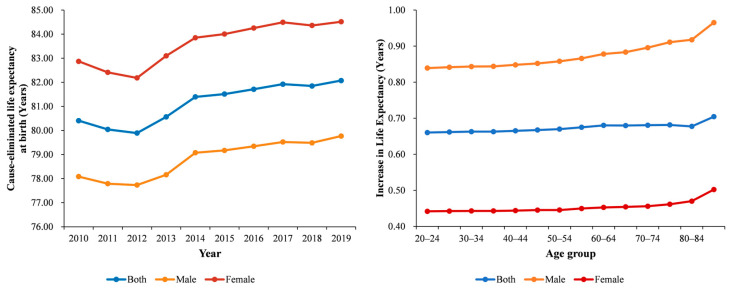
The trends of cause-eliminated life expectancy and increase in life expectancy by sex.

Figure 3 showed the trends of cause-eliminated life expectancy and increase in life expectancy by sex. In Wuhan, life expectancy at birth for both sexes increased by 1.63 years, rising from 79.79 years in 2010 to 81.42 years in 2019. Additionally, the life expectancy for females was consistently higher than that for males. During the same period, the CELE at birth for both sexes increased by 1.67 years, from 80.40 years in 2010 to 82.07 years in 2010. If we eliminate COPD, life expectancy would increase by 0.66–0.70 years for both sexes, with an increase of 0.84–0.97 years for males and 0.44–0.50 years for females, indicating that males, especially older males, are more sensitive to COPD.

**Figure 4:**
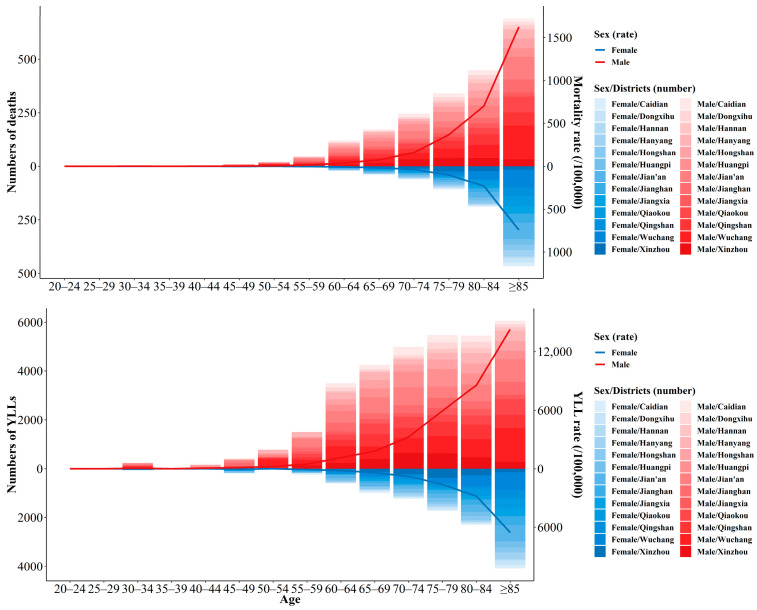
Deaths, YLLs, mortality, and YLL rate for COPD by age and sex in 2019.

Deaths and YLLs related to COPD by age and sex in 2019 are shown in Figure 4. In 2019, the COPD deaths increased synchronously with age in males and females, with the peak appearing at ≥85 group, and more age-specific deaths occurred in males. Correspondingly, the age-specific mortality rate was higher in males than in females, with a sharp increase at the age of 65 years and older. The age-specific number of YLLs for COPD showed the similar pattern with that of deaths, and the increase in YLLs was more obvious after the age of 60–64 years. The age-specific number of YLLs was much higher in males than that in females; correspondingly, the age-specific YLL rate in males was higher.

### 3.3. The Age, Period, and Cohort Effects on COPD Mortality and YLL Rate

**Figure 5:**
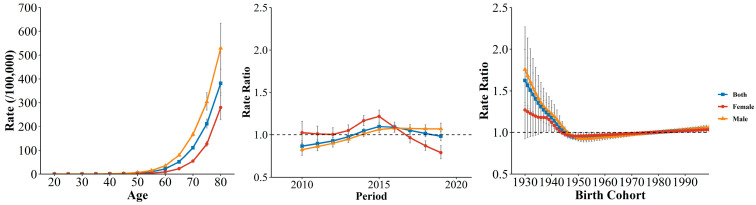
The age, period, and cohort effects on COPD mortality by sex in Wuhan.

Figure 5 illustrated the age effects on sex-specific COPD mortality. After controlling for period and cohort effects, the COPD mortality exhibited an exponential distribution with age, especially after 60 years old. Additionally, compared to females, males showed a more pronounced increasing trend, and this sex disparity in mortality gradually increased with age. The estimated period RRs first increased and then decreased for both sexes. Females showed the significant downward trends in period RRs after 2015, with the lowest point appearing in 2019. However, the period RRs of males showed the upward trends, increasing from 0.83 to 1.07, although it has stagnated during a recent period. The improvements in COPD risk were more significant for females in Wuhan. In terms of estimated cohort RRs, the cohort RRs for both sexes exhibited a decreasing trend followed by a slight increase. Considering the whole birth cohort cycle, the birth cohort risk has improved since the 1930s cohort, especially for males and for those born before the 1960s. The cohort RRs remained the lowest for females throughout the whole birth cohort cycle.

**Figure 6:**
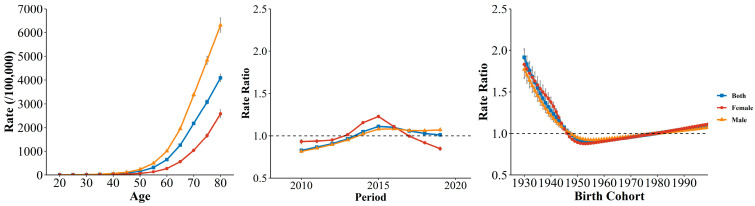
The age, period, and cohort effects on COPD YLL rate by sex in Wuhan.

Additionally, similar changing patterns were observed in YLL rates for COPD. The age curves rose exponentially with age, and males were higher than females among the whole age groups. Period RRs also experienced an increase followed by a decrease, with the turning point being 2015. Females performed better in improving period RR than males. The similar decreasing and then increasing trends were also observed in the cohort RR (Figure 6).

## 4. Discussion

Our study provided an updated overview of the temporal trends and spatial distributions of age-standardized mortality and age-standardized YLL rates in COPD, based on 10 years of data from the Wuhan cause of death surveillance system. Additionally, the APC model was adopted to explore the age, period, and cohort effects on COPD mortality and YLL rate independently by sex.

Recently, the ASMR and ASYR for COPD in Wuhan have shown decreasing trends, especially after 2015. In 2019, the ASMR and ASYR in Wuhan was 24.74 (23.82, 25.72) and 404.20 (400.29, 408.16), respectively. According to GBD 2019, the global ASMR and ASYR for COPD both showed decreasing trends during the study period, with China’s ASMR decreasing from 99.95 (89.74, 110.10) in 2010 to 65.20 (55.51, 80.09) in 2019 and ASYR from 1344.06 (1220.12, 1478.65) in 2010 declined to 862.37 (736.42, 1,053.64) in 2019 [15]. The burden of COPD deaths in Wuhan is lower than the national average level to a certain extent. Additionally, sex and regional disparities were observed in Wuhan. During the study period, the death burden of COPD was more severe in males than in females, with higher ASMR and ASYR in males. Previous studies have also shown that COPD posed a greater threat to males [8,16]. Possibly because males have a higher prevalence of smoking and may encounter more COPD risk factors in their occupational activities, such as mineral dusts (quartz, asbestos, talc), inorganic dusts, metal dusts (iron, tin, aluminum) and cereals. Additionally, females may have better compliance to treatment, which also directly affects the risk of death from COPD [17,18]. In addition, dietary habits (adequate intake of fruits and vegetables, low salt diet, etc.), health awareness, socioeconomic status, and physical activity preferences were also important reasons for the sex differences in the death risk from COPD [19,20].

Qingshan District, a heavy industry area of Wuhan, has the highest ASMR and ASYR in 2019. Air pollution is an important factor in COPD. During the period between 2014 and 2019, the daily average concentrations of PM 2.5 (59.03 μg/m^3^), PM 10 (90.48 μg/m^3^) and NO_2_ (48.84 μg/m^3^) were 5.90, 4.52 and 1.22 times higher than the WHO Air Quality Guidelines (10, 20, and 40 μg/m^3^, respectively) [21]. Previous studies have shown the harmful effects of air pollution on health, particularly on respiratory health [22,23]. Meanwhile, meta-analysis also showed that ambient air pollution contributes to an increased incidence, prevalence, hospitalizations and mortality of COPD [24,25]. Particulate matter may cause oxidative stress on lung cells by presenting or stimulating the cellular production of reactive oxygen species (ROS) and inflammatory factors, which were involved in lung tissue damage [26,27]. In addition, studies found that smokers were more susceptible to PM 2.5 pollution. PM 2.5 and cigarette smoke may act synergistically on the development of COPD [28]. However, with the promotion of the use of clean energy in Wuhan, the recent air pollution in Wuhan would be improved in the future.

In addition, we used the APC model to decompose age, period and birth cohort effects to reveal the potential influencing factors on trends in COPD death burden. Both the ASMR and ASYR for COPD in Wuhan showed obvious age effects. Once the age exceeds 60, the ASMR and ASYR will increase exponentially, which implies the influence of population aging on COPD. Aging is the progressive degeneration of tissues, leading to decreased immunity and lung function [29]. Studies have shown that after the age of 30, there is a decline in lung function and structural dysfunction with increasing age [30,31]. Simultaneously, the effectiveness of the lung defense mechanism diminishes with aging, increasing the risk of lung infection [32]. When the body’s anti-inflammatory and antioxidant mechanisms fail to protect against the damage caused by sustained low-level inflammation and increased ROS, the accumulation of risk factors such as smoking and air pollution may promote the occurrence and development of COPD in the elderly. Previous studies have shown that since Wuhan was listed as an aging city in 1993, the average annual growth rate of the elderly population over 60 in Wuhan has reached approximately 3.00% [33]. By the end of 2017, the number of elderly people over 60 years old in Wuhan accounted for 20.95% of the total population [34,35]. In 2019, the average life expectancy of the Wuhan population is 81.42 years, higher than the national average level (77.30). With the deepening of aging, the aging of population will become an obstacle to the improvement of COPD burden.

The period effects represent changes in medical technology, economy and culture specific to a specific period. Period RRs for the Wuhan population generally tended to rise and then fall between 2010 and 2019, with females showing a clear downward trend after 2015. Males, however, maintained an upward trend throughout the study period, although the growth trend has stagnated in recent years. The significant downward trend observed in this study may be mainly due to increased health resources, improved socioeconomic status, and increased health awareness among people. During the study period, China has gradually expanded the coverage of health insurance [8,36]. The enhanced diagnosis and treatment techniques, better access to basic public health services, the new rural cooperative medical system, and the reform of COPD healthcare costs have promoted the improvement of COPD death burden in Wuhan [37]. However, the smoking situation in Wuhan is still very serious. Two surveys on the smoking prevalence in Wuhan showed little improvement in the smoking rate among the population over the age of 15 in Wuhan (26.0% in 2015 vs. 25.8% in 2016), with smoking prevalence of male being 48.9% in 2015 and 49.1% in 2016 [38,39]. Considering the relatively serious air pollution in Wuhan, the synergistic effect between smoking and air pollution could be a potential reason for the increasing trend of period RR in males in Wuhan [28,40]. Therefore, continuing to enhance the healthcare policy and healthcare coverage system and implementing a more equitable healthcare resource allocation strategy may help to significantly reduce the death burden of COPD in the Wuhan population.

Cohort effects refer to the long-term trend of disease mortality of those born around the same time, which may be related to lifestyle, external environment, and risk factor exposure. The cohort effect of COPD death burden in the Wuhan population was found to be generally decreasing, reflecting that, in a more mature medical, educational, and social environment, the later birth cohort is better able to avoid exposure to risk factors associated with COPD death (e.g., smoking, occupational exposure, poor lifestyle and dietary habits). Economic development and the related environmental improvement may be the main reason for the declining trend of cohort RR in Wuhan. Previous studies have shown that environmental factors in utero and early childhood have a profound effect on the development of lung function [41,42]. Many different prenatal factors can adversely affect fetal lung growth [43], including nicotine and tobacco smoke exposure and the effect of maternal stress on the fetal immune response [44]. Therefore, better maternal and perinatal care and improvements in the early childhood environment would help to reduce the death risk from COPD in adulthood. Additionally, a better childhood environment is associated with a reduction in early infections and a lower risk of COPD in adulthood [45]. Meanwhile, better nutrition level and a better awareness of COPD-related knowledge may also play an important role in reducing COPD burden in future generations.

There are several limitations to our study. We excluded the population aged 0–20 years and 85 years or older, because COPD deaths data for the under-20 age group was few in Wuhan, and the population over 84 years old was assigned as one age group, which did not meet the requirements of the APC model. Additionally, the APC model was based on the population level, so ecological fallacy might occur, and the interpretation of the study might not apply to the individual level. In this study, we focused on the spatial–temporal patterns of COPD death burden in Wuhan, and in subsequent studies, we will calculate the prevalence and disability-adjusted life years to provide more different perspectives on the COPD burden in Wuhan.

## 5. Conclusions

Recently, the death burden of COPD in Wuhan has improved to a certain extent, especially after 2015, with the decreasing trends in both ASMR and ASYR. Additionally, the death risk ratios of COPD have declined overall with time, particularly among the generations born after than 1960s. However, sex and regional differences still existed. COPD posed a greater threat to elderly, especially males. Meanwhile, in 2019, the death burden of COPD was relatively higher in the Qingshan and Jian’an districts. Considering the population aging, life expectancy extension, relatively high smoking rate and serious air pollution, COPD remains a crucial public health issue in Wuhan, and public health managers should continue to execute more targeted programs to lessen the death burden of COPD.

## Figures and Tables

**Table 1:** The ASMR, ASYR, and EAPCs for COPD from 2010 to 2019.

Characteristics	ASMR Per 100,000 No. (95% UI)		EAPC No. (95% CI)	ASYR Per 100,000 No. (95% UI)		EAPC No. (95% CI)
	2010	2019		2010	2019	
Overall	25.65 (24.52, 26.82)	24.74 (23.82, 25.72)	0.31 (−1.14, 1.79)	420.25 (415.55, 424.98)	404.20 (400.29, 408.16)	0.32 (−1.24, 1.92)
Sex						
Male	36.96 (34.95, 39.06)	39.6 (37.87, 41.44)	1.06 (0.13, 2.00)	610.8 (602.51, 619.17)	659.07 (909.03, 651.77)	1.21 (0.12, 2.31)
Female	16.32 (15.13, 17.59)	12.18 (11.34, 13.16)	−1.67 (−4.26, 0.98)	252.01 (247.08, 257.03)	177.36 (319.25, 173.92)	−2.14 (−4.95, 0.74)
Regions						
Caidian	16.52 (12.87, 21.01)	21.7 (17.87, 26.77)	8.39 (−0.40, 17.95)	291.83 (275.33, 309.12)	364.39 (347.81, 382.07)	8.28 (−1.07, 18.52)
Dongxihu	49.27 (39.44, 60.92)	30.66 (24.96, 38.58)	−0.50 (−7.31, 6.82)	773.02 (732.51, 815.29)	449.48 (426.53, 474.33)	−2.15 (−9.11, 5.35)
Hannan	83.45 (70.05, 98.79)	20.19 (15.26, 28.04)	−10.57 (−14.14, −6.87)	1265.76 (1211.04, 1322.39)	278.64 (259.08, 300.56)	−10.97 (−14.62, −7.15)
Hanyang	30.36 (25.4, 36.20)	26.05 (22.32, 31.73)	−0.08 (−2.68, 2.6)	530.34 (508.55, 552.97)	433.99 (417.72, 451.79)	−0.34 (−2.73, 2.12)
Hongshan	34.90 (30.23, 40.36)	16.79 (14.38, 19.69)	−5.84 (−10.19, −1.28)	574.6 (554.79, 595.17)	269.12 (258.75, 279.95)	−6.34 (−10.8, −1.65)
Huangpi	15.56 (13.16, 18.29)	19.21 (16.82, 22.01)	3.92 (−0.39, 8.43)	269.28 (258.81, 280.09)	329.62 (319.19, 340.42)	3.97 (−0.52, 8.65)
Jiang’an	24.50 (21.32, 28.2)	29.76 (26.72, 33.93)	2.23 (−1.32, 5.9)	401.61 (387.79, 415.95)	501.97 (488.53, 516.34)	2.77 (−0.49, 6.15)
Jianghan	29.42 (24.92, 34.72)	26.78 (23.18, 32.11)	−2.84 (−5.47, −0.13)	485.59 (466.03, 505.94)	457.15 (440.98, 474.71)	−2.58 (−5.03, −0.06)
Jiangxia	36.37 (31.07, 42.36)	21.78 (18.21, 26.1)	−4.02 (−7.2, −0.73)	602.57 (580, 625.82)	323.22 (308.67, 338.49)	−4.96 (−8.71, −1.06)
Qiaokou	34.93 (30.7, 39.8)	26.38 (23.05, 31.22)	−3.39 (−4.49, −2.27)	502 (485.05, 519.58)	433.38 (418.53, 449.46)	−2.98 (−4.8, −1.12)
Qingshan	37.02 (31.47, 43.37)	31.27 (27.53, 36.96)	3.44 (−2.3, 9.53)	573.58 (551.53, 596.4)	523.35 (506.75, 541.47)	4.66 (−1.47, 11.17)
Wuchang	15.42 (13.1, 18.13)	27.67 (25.1, 30.89)	4.62 (1.93, 7.37)	241.96 (232.3, 252.01)	452.72 (441.42, 464.58)	4.90 (1.58, 8.32)
Xinzhou	9.48 (7.45, 11.92)	23.29 (20.44, 26.59)	4.38 (−4.08, 13.58)	191.18 (181.62, 201.14)	391.16 (379.11, 403.65)	3.15 (−4.90, 11.88)

## Data Availability

Not applicable.

## Supplementary Materials

The following supporting information can be downloaded at: https://www.mdpi.com/article/10.3390/ijerph191710685/s1, Table S1: Wald Chi Square tests for estimable functions in the APC model.

## References

[1] Diem G., Brownson R.C., Grabauskas V., Shatchkute A., Stachenko S. (2016). Prevention and control of noncommunicable diseases through evidence-based public health: Implementing the NCD 2020 action plan. Glob. Health Promot..

[2] GBD 2015 Mortality and Causes of Death Collaborators (2016). Global, regional, and national life expectancy, all-cause mortality, and cause-specific mortality for 249 causes of death, 1980–2015: A systematic analysis for the Global Burden of Disease Study 2015. Lancet.

[3] Adeloye D., Chua S., Lee C., Basquill C., Papana A., Theodoratou E., Nair H., Gasevic D., Sridhar D., Campbell H. (2015). Global and regional estimates of COPD prevalence: Systematic review and meta-analysis. J. Glob. Health.

[4] GBD 2017 Disease and Injury Incidence and Prevalence Collaborators (2018). Global, regional, and national incidence, prevalence, and years lived with disability for 354 diseases and injuries for 195 countries and territories, 1990–2017: A systematic analysis for the Global Burden of Disease Study 2017. Lancet.

[5] GBD 2019 Diseases and Injuries Collaborators (2020). Global burden of 369 diseases and injuries in 204 countries and territories, 1990–2019: A systematic analysis for the Global Burden of Disease Study 2019. Lancet.

[6] Yang G., Wang Y., Zeng Y., Gao G.F., Liang X., Zhou M., Wan X., Yu S., Jiang Y., Naghavi M. (2013). Rapid health transition in China, 1990–2010: Findings from the Global Burden of Disease Study 2010. Lancet.

[7] Zhou M., Wang H., Zeng X., Yin P., Zhu J., Chen W., Li X., Wang L., Wang L., Liu Y. (2019). Mortality, morbidity, and risk factors in China and its provinces, 1990–2017: A systematic analysis for the Global Burden of Disease Study 2017. Lancet.

[8] Liu W., Wang W., Liu J., Liu Y., Meng S., Wang F., Long Z., Qi J., You J., Lin L. (2021). Trend of Mortality and Years of Life Lost Due to Chronic Obstructive Pulmonary Disease in China and Its Provinces, 2005–2020. Int. J. Chronic Obstr. Pulm. Dis..

[9] Mubarik S., Yu Y., Wang F., Malik S.S., Liu X., Fawad M., Shi F., Yu C. (2022). Epidemiological and sociodemographic transitions of female breast cancer incidence, death, case fatality and DALYs in 21 world regions and globally, from 1990 to 2017: An Age-Period-Cohort Analysis. J. Adv. Res..

[10] Sun L., Zhou Y., Zhang M., Li C., Qu M., Cai Q., Meng J., Fan H., Zhao Y., Hu D. (2022). Association of Major Chronic Noncommunicable Diseases and Life Expectancy in China, 2019. Healthcare.

[11] Yang Y. (2008). Trends in U.S. adult chronic disease mortality, 1960–1999: Age, period, and cohort variations. Demography.

[12] Ko C.W. (2014). Age-Period-Cohort Analysis: New Models, Methods, and Empirical Applications. J. Am. Stat. Assoc..

[13] Wang P., Xu C., Yu C. (2014). Age-period-cohort analysis on the cancer mortality in rural China: 1990–2010. Int. J. Equity Health.

[14] Jinhong C., Eshak E.S., Keyang L., Krisztina G., Zhiming L., Chuanhua Y. (2019). Age-Period-Cohort Analysis of Stroke Mortality Attributable to High Sodium Intake in China and Japan. Stroke.

[15] Yin P., Wu J., Wang L., Luo C., Ouyang L., Tang X., Liu J., Liu Y., Qi J., Zhou M. (2022). The Burden of COPD in China and Its Provinces: Findings from the Global Burden of Disease Study 2019. Front. Public Health.

[16] Wang C., Xu J., Yang L., Xu Y., Zhang X., Bai C., Kang J., Ran P., Shen H., Wen F. (2018). Prevalence and risk factors of chronic obstructive pulmonary disease in China (the China Pulmonary Health [CPH] study): A national cross-sectional study. Lancet.

[17] Kokturk N., Kilic H., Baha A., Lee S.D., Jones P.W. (2016). Sex Difference in Chronic Obstructive Lung Disease. Does it Matter? A Concise Review. COPD J. Chronic Obstr. Pulm. Dis..

[18] Xie L., Qian Y., Liu Y., Li Y., Jia S., Yu H., Wang C., Qian B., Bao P. (2020). Distinctive lung cancer incidence trends among men and women attributable to the period effect in Shanghai: An analysis spanning 42 years. Cancer Med..

[19] Matera M.G., Ora J., Calzetta L., Rogliani P., Cazzola M. (2021). Sex differences in COPD management. Expert Rev. Clin. Pharmacol..

[20] Siegfried J.M. (2022). Sex and Gender Differences in Lung Cancer and Chronic Obstructive Lung Disease. Endocrinology.

[21] Yan Y., She L., Guo Y., Zhao Y., Zhang P., Xiang B., Zeng J., Yang M., Wang L. (2021). Association between ambient air pollution and mortality from chronic obstructive pulmonary disease in Wuhan, China: A population-based time-series study. Environ. Sci. Pollut. Res. Int..

[22] Schikowski T., Mills I.C., Anderson H.R., Cohen A., Hansell A., Kauffmann F., Krämer U., Marcon A., Perez L., Sunyer J. (2014). Ambient air pollution: A cause of COPD?. Eur. Respir. J..

[23] Liu S., Zhou Y., Liu S., Chen X., Zou W., Zhao D., Li X., Pu J., Huang L., Chen J. (2017). Association between exposure to ambient particulate matter and chronic obstructive pulmonary disease: Results from a cross-sectional study in China. Thorax.

[24] Li M.-H., Fan L.-C., Mao B., Yang J.-W., Choi A.M.K., Cao W.-J., Xu J.-F. (2016). Short-term Exposure to Ambient Fine Particulate Matter Increases Hospitalizations and Mortality in COPD: A Systematic Review and Meta-analysis. Chest.

[25] Atkinson R.W., Kang S., Anderson H.R., Mills I.C., Walton H.A. (2014). Epidemiological time series studies of PM2.5 and daily mortality and hospital admissions: A systematic review and meta-analysis. Thorax.

[26] Tao F., Gonzalez-Flecha B., Kobzik L. (2003). Reactive oxygen species in pulmonary inflammation by ambient particulates. Free Radic. Biol. Med..

[27] Liu C.-W., Lee T.-L., Chen Y.-C., Liang C.-J., Wang S.-H., Lue J.-H., Tsai J.-S., Lee S.-W., Chen S.-H., Yang Y.-F. (2018). PM(2.5)-induced oxidative stress increases intercellular adhesion molecule-1 expression in lung epithelial cells through the IL-6/AKT/STAT3/NF-κB-dependent pathway. Part. Fibre Toxicol..

[28] Zhao J., Li M., Wang Z., Chen J., Zhao J., Xu Y., Wei X., Wang J., Xie J. (2019). Role of PM(2.5) in the development and progression of COPD and its mechanisms. Respir. Res..

[29] Easter M., Bollenbecker S., Barnes J.W., Krick S. (2020). Targeting Aging Pathways in Chronic Obstructive Pulmonary Disease. Int. J. Mol. Sci..

[30] Lahousse L., Seys L.J.M., Joos G.F., Franco O.H., Stricker B.H., Brusselle G.G. (2017). Epidemiology and impact of chronic bronchitis in chronic obstructive pulmonary disease. Eur. Respir. J..

[31] Fragoso C.A. (2016). Epidemiology of Chronic Obstructive Pulmonary Disease (COPD) in Aging Populations. COPD J. Chronic Obstr. Pulm. Dis..

[32] Brandsma C.A., de Vries M., Costa R., Woldhuis R.R., Königshoff M., Timens W. (2017). Lung ageing and COPD: Is there a role for ageing in abnormal tissue repair?. Eur. Respir. Rev..

[33] Peng J., Xu X., Jiang L. (2012). A Preliminary Study on Planning Response to Population Aging in Wuhan City. Chin. Overseas Archit..

[34] Zhang L. (2015). Impact of Population Aging on Wuhan City Pension Fund. Ploneering Sci. Technol. Mon..

[35] Li J., Zhao H. (2018). Wuhan City Promotes “Five Combinations” of National Education on Population Aging. China Soc. Work..

[36] Zhu B., Wang Y., Ming J., Chen W., Zhang L. (2018). Disease burden of COPD in China: A systematic review. Int. J. Chronic Obstr. Pulm. Dis..

[37] Bai J., Zhao Y., Yang D., Ma Y., Yu C. (2022). Secular trends in chronic respiratory diseases mortality in Brazil, Russia, China, and South Africa: A comparative study across main BRICS countries from 1990 to 2019. BMC Public Health.

[38] Lu Y., Li J., Huang Y., Li Y., Zhang L., Peng L., Mei X., Chen M., Deng Z. (2018). Survey on tobacco prevalence among urban residents in Wuhan City. Chin. J. Health Educ..

[39] Wu C., Deng Z. (2018). Epidemiologic status survey on tobacco epidemic among residents aged 15 and above of Wuhan in 2015. Mod. Prev. Med..

[40] Ni L., Chuang C.C., Zuo L. (2015). Fine particulate matter in acute exacerbation of COPD. Front. Physiol..

[41] Cho S.J., Stout-Delgado H.W. (2020). Aging and Lung Disease. Annu. Rev. Physiol..

[42] Bush A. (2016). Lung Development and Aging. Ann. Am. Thorac. Soc..

[43] Rusconi F., Galassi C., Forastiere F., Bellasio M., De Sario M., Ciccone G., Brunetti L., Chellini E., Corbo G., La Grutta S. (2007). Maternal complications and procedures in pregnancy and at birth and wheezing phenotypes in children. Am. J. Respir. Crit. Care Med..

[44] Lee A., Wright R.J. (2016). Prenatal stress and childhood asthma risk: Taking a broader view. Eur. Respir. J..

[45] Wen H., Xie C., Wang L., Wang F., Wang Y., Liu X., Yu C. (2019). Difference in Long-Term Trends in COPD Mortality between China and the U.S.; 1992–2017: An Age-Period-Cohort Analysis. Int. J. Environ. Res. Public Health.

