# Supplementary material for: Secular Trends of Mortality and Years of Life Lost Due to Chronic Obstructive Pulmonary Disease in Wuhan, China from 2010 to 2019: Age-Period-Cohort Analysis
Source: Int J Environ Res Public Health. 2022 Aug 27;19(17):10685. doi: 10.3390/ijerph191710685 (PMC9518558; doi:10.3390/ijerph191710685)
Supplement: Supplementary file 1 [file ijerph-19-10685-s001.zip › ijerph-1890692-supplementary.pdf]

Supplemental Table S1. Wald Chi Square tests for estimable functions in the APC model.

Model	Mod. dev	*P* value
**Total**		
1. Age	231.54	
2. Age-drift	223.88	**0.01**
3. Age-cohort	208.18	**0.00**
4. Age-period	175.59	**0.00**
5. Age-period-cohort	153.48	**0.00**
**Male**		
1. Age	179.89	
2. Age-drift	178.37	0.22
3. Age-cohort	163.25	**0.00**
4. Age-period	166.37	**0.00**
5. Age-period-cohort	145.81	**0.00**
**Female**		
1. Age	275.47	
2. Age-drift	227.69	**0.00**
3. Age-cohort	217.27	**0.02**
4. Age-period	168.32	**0.00**
5. Age-period-cohort	163.13	**0.00**
